# Malnutrition in Spinal Cord Injury: More Than Nutritional Deficiency

**DOI:** 10.4021/jocmr924w

**Published:** 2012-07-20

**Authors:** Yannis Dionyssiotis

**Affiliations:** Medical Department, Rehabilitation Center Amyntaio, General Hospital of Florina, 2nd km National Road Amyntaio-Thessaloniki, 53200, Amyntaio, Florina, Greece. Email: yannis_dionyssiotis@hotmail.com

**Keywords:** Spinal cord injury, Malnutrition, Obesity, Physiopathology, Rehabilitation

## Abstract

Denervation of the spinal cord below the level of injury leads to complications producing malnutrition. Nutritional status affects mortality and pathology of injured subjects and it has been reported that two thirds of individuals enrolled in rehabilitation units are malnourished. Therefore, the aim should be either to maintain an optimal nutritional status, or supplement these subjects in order to overcome deficiencies in nutrients or prevent obesity. This paper reviews methods of nutritional assessment and describes the physiopathological mechanisms of malnutrition based on the assumption that spinal cord injured subjects need to receive adequate nutrition to promote optimal recovery, placing nutrition as a first line treatment and not an afterthought in the rehabilitation of spinal cord injury.

## Introduction

During the last 50 years the medical world has become aware of the effectiveness of early, aggressive nutrition in reducing complications in spinal cord injury (SCI). Specific dietary approaches are indicated for this population to achieve optimal nutritional status [1, 2]. Nevertheless, nutritional deficiency in a hospital setting is still a common issue, despite the advances in medical and nutritional science. Surveys show that 40-50% of patients admitted to hospitals are at risk of nutritional deficiency and up to 12% are severely malnourished [3, 4]. On the other site, studies report a variable prevalence of obesity from 40 to 66% in persons with SCI completing the spectrum of malnutrition [5-8]. In subjects with SCI particular attention should be paid to the metabolic differences in the nutritional status in order to optimize the medical and neurological benefits. After the injury, well documented hyper catabolic responses may lead to deleterious effects such as loss of lean body mass, obesity, increased susceptibility to infections, and reduced wound healing [5, 9]. The paralysis and loss of function that usually occur after a SCI produce additional metabolic and nutritional deficiencies with tetraplegics being at higher risk of malnutrition than paraplegics [10, 11]. SCI subjects lose muscle mass and bone and gain fat mass especially below the level of injury. Unwanted weight gain should be prevented because induces the risk for diseases such as diabetes, coronary heart disease and dyslipidaimias in this population [9]. According to the above the term malnutrition includes not only under nutrition but also over nutrition/obesity. The optimal nutritional assessment and management of the spinal cord injured subject can minimize the complications associated with acute traumatic injury and long-term rehabilitation [12]. This paper reviews methods of nutritional assessment and describes the physiopathological mechanisms of malnutrition in SCI subjects.

## Nutritional Assessment

No single parameter can continually assess the nutritional status or provide the effect of diet in preventing and treating complications. For an initial assessment of nutritional status serial measurements to assess trends over time and then monitor the response to a dietary intervention may be useful. The proposed assessments should be interpreted collectively including the examination of possible factors that contribute to the nutritional status, such as age, sex, over-or under-hydration, interactions between drugs-food, metabolic stress, infection, and the existence of other diseases [13].

### Diet history

Adequate intake of nutrients is intercepted by many factors, such as difficulty in chewing and swallowing, difficulty in obtaining and preparing food, immobility, negligence, depression, and anorexia. Assistance during meals may be needed if SCI subjects are not in a position to feed by themselves, but using adaptive equipment they can become more independent [8]. Moreover, gastrointestinal function is compromised: gastric dilatation and paralytic ileus occurs often, although the intestinal activity usually returns within the first week after injury. For patients with dysphagia or stricture of the esophagus soft food usually improves the nutrient intake. Constipation can be prevented with adequate dietary fiber and fluids while rates of gastric evacuation are normal, regardless of level or complete or incomplete nature of the lesion [14].

### Nutritional requirements

The provision of energy and nutritional requirements is a very important factor for patient management. Malnutrition, in this case under or over nutrition, can lead to muscle loss, atrophy of the lining of the intestine, immunochemical reduction, poor wound healing and fluid overload, hyperglycemia, high levels of urea nitrogen in blood, high triglycerides, elevated liver enzymes, respiratory exhaustion due to increased production of CO_2_, and difficulty weaning from the oxygen, respectively. The assessment of nutritional requirements includes not only calculations but also the opinion of an expert clinician in order to assess the clinical and morphometric data before applying the equations that provide the energy and protein requirements [15].There have been several methods for predicting energy expenditure (EE); the components and the methods for its determination and estimation, summarizing their main advantages and limitations have been recently reviewed [16]. However, because of various confusing factors such as infections and sepsis, hyper nutrition supportive nutritional diets, clinical procedures, postoperative medications, and changes in body weight such as sarcopenia, obesity, amputations and significant weight loss, the prediction equations can be complex and invalid [17]. Basic energy expenditure (BEE) is estimated by the equation of Harris-Benedict (HBE), while BEE is calculated by sex with the following formula: Men: 66.5 + 13.8 (wt) + 5.0 (ht) − 6.8 (age), Women: 655.1 + 9.6 (wt) + 1.8 (ht) − 4.7 (age), where wt is the weight in kilograms and ht is height in meters, is considered as the standard criterion in predicting caloric requirements of patients in the acute phase of the disease for many years, although it may overestimate energy needs [18, 19]. Predicted energy expenditure (PEE) is calculated as follows: (BEE) x (activity rate) x (the agent of injury), while activity rate (an activity factor for bed rest) and agent of injury (an injury factor for major trauma) are 1.2 and 1.6, respectively [20].

### Assessment of subjects in the clinical setting

Patients admitted in the hospital should be examined for actual or potential occurrence of malnutrition because of an unintentional weight loss or gain. In the clinic this examination includes measurements of body weight depicting a loss of more than 10% of normal body weight within 6 months or loss of more than 5% of usual body weight within 1 month or 20% more or less than ideal body weight (IBW), calculation of body mass index (BMI) < 18, depletion of visceral protein (serum albumin < 3.5 g/dl, serum transferrin < 200 mg/dl, serum cholesterol < 160 mg/dl, serum pre-albumin < 15 mg/ml, creatinine height index (CHI) < 75% (measured by 24-hour creatinine excretion, which is typically associated with muscle mass of the patient as an indicator of malnutrition, especially in young men), and the presence of diet modifications (patient receives total parenteral nutrition (TPN) or enteral nutrition (EN), inadequate food intake due instructions for stopping any food by mouth (NPO), liquid diet, disorders of absorption, reduced swallowing capacity, increased metabolic needs, gastrointestinal disturbances (nausea, vomiting, diarrhea, constipation). Unintentional weight gain is an increase in body weight that occurs when a person takes in more calories than the body needs or uses [21].

### Anthropometric evaluation

For able bodied persons the World Health Organization (WHO) advocates use of BMI as a population-level indicator of obesity which is not a direct measure of body fat, but a more accurate indicator of overweight and obesity than relying on weight alone. BMI is calculated using the equation weight (Kg)/height (m^2^), which is a very practical and useful measure that allows the easy determination of categories of weight status. In able bodied subjects overweight is defined as a BMI of 25 - 29.9 kg/m^2^ and obesity as a BMI of ≥ 30.0 kg/m^2^ (Table 1). In chronic spinal cord injured patients it measuring fat with BMI is not enough to determine subject’s percentage of fat in the body (see discussion) [9, 22].

**Table 1:** Classifications Based on the Weight for BMI and Obesity Category [3]

Classification	BMI (kg/m^2^)	Obesity Category
Underweight	<18.5	-
Normal	18.5-24.9	-
Overweight	25.0-29.9	-
Obesity	30.0-34.9	I
Moderate obesity	35.0-39.9	II
Extreme obesity	> 40.0	III

Anthropometric standards such as the ideal body weight (IBW), the triceps skin fold thickness and the middle arm circumference which are common tools for assessment of nutrition may not be valid for patients with spinal cord injury due to water changes, atrophy of muscles because of immobility, increased body fat, and the inevitable weight loss beyond the normal. Patients’ early weight loss is mainly due to loss of muscle rather than fat which bias the results of validity. In chronic paraplegics, the ideal weight has been estimated to be 4.5 to 6.5 kg below their respective controls finding which is in line with our recently published results [9]. Indeed, height and weight measurements are the key elements in nutritional assessment. The IBW is determined by the height. No matter which method of calculation is used, the IBW should be adjusted for body type (frame sizes: small - IBW 10% reduction, middle size - no changes required, large size - IBW increased by 10%) and spinal cord injury (paraplegia - decrease IBW by 10-15%, tetraplegia - by 15-20%, respectively). The weight in admission is probably the most reliable measure of weight in determining the actual body weight (ABW) of the patient because is unreliable postoperatively or during an acute illness due to administration of fluids or due to edematous condition. As a chronic index, one can assume that the weight gain or loss is associated with an increase or decrease in lean body mass. To determine the weight which should be used on the nutritional calculations, first % IBW should be calculated through the equation: % IBW = actual body weight (actual body weight, ABW / ideal body weight (IBW) x 100. If the actual body weight (ABW) is less than IBW, use ABW, to define the nutritional requirements, if is greater than IBW, but less than 120%, it is necessary to determine nutritional needs using the adjusted relationship of body weight in the calculation needs: IBW + (ABW - IBW x 0.25). The nutritional status of patients can be categorized according to their ABW as a percentage of IBW as follows: over 200% of IBW (pathologic obesity), over 150% of IBW (obese), more than 120% IBW (overweight), 100% of IBW +/- 10% (normal), 80-90% of IBW (mildly malnourished), 70-80% of IBW (moderately malnourished), less than 70% of IBW (severe nutritional deficiency-malnutrition), respectively [3]. In generally anthropometric measures in spinal cord injured persons tend to underestimate fat percentage when compared with able bodied individuals.

### Biochemical measurements

As with the visceral and somatic visceral proteins, non-dietary factors (i.e. blood loss, chronic infections, and fluid overload) should be considered as potential reasons for the reduction of serum concentrations [3]. Proteins are essential for tissue growth, maintenance and rebuilding their synthesis of hormones, enzymes, antibodies and cells transport molecules. If excess protein or metabolized for energy or stored as fat. The recommendations for protein intake in patients with spinal cord injury vary with respect to acute or chronic phase of the lesion and the presence of decubitus ulcers or not.

Specific proteins (albumin, transferrin, and pre-albumin) are biochemical indicators used for assessing nutritional status [23]. The level of serum albumin is not a definitive measure of visceral protein status, but reflects the complex relationship between synthesis, degradation, and distribution. Given the long half-life of 21 days, serum albumin cannot be effectively used for monitoring the acute response to nutritional therapy. Therefore, albumin levels should be included in the initial profile for food control and monitoring purposes during hospitalization for measuring trends of visceral protein or as an indicator of chronic nutritional status (Table 2). Beside this limitation there are many non-dietary factors that reduce the levels of albumin, regardless of nutritional status (inadequate composition: acute stress, hypoxia, impaired digestion, as in malabsorption, modified status as edematous fluid status and fluid overload, chronic loss of protein).

**Table 2:** Basic Levels of Albumin and Nutritional Status Distribution

Albumin (g/dl)	3.5 - 5	3 - 3.5	< 3.5	< 3	< 2.5
Nutritional status	normal	Point that dietary intervention should be revised or adjusted.	Associated with poor outcome of surgery, rising costs of hospitalization and prolonged stay in ICU.	Severe malnutrition	Increased morbidity and mortality.

Due to the lower half-life (8 - 9 days) and the smaller size as a constituent body, transferrin is a better indicator of nutritional status of visceral protein from albumin. Normal levels of transferrin are between 200 - 400 mg/dl, and 150 mg/dl are considered nutritionally decision point or a point where nutritional support should be revised or adjusted. The transferrin levels are reduced in impaired synthesis as chronic infections, increased secretion, fluid overload, increased iron stores and increased in reduced iron stores as iron deficiency anemia and chronic blood loss, increased protein synthesis on estrogen therapy and oral contraception and dehydration. The serum concentration of transferrin is approximately 0.8 times the total iron binding capacity (TIBC). If direct measurement of transferrin is not possible due to the high cost and limited availability of equipment required, the level of transferrin can be easily calculated from TIBC, using the following formula: TIBC x 0.8 − 43 = transferrin [24].

The third protein biochemical indicator is pre-albumin, which has very short half-life (2 days), making it an excellent nutritional index and due to this reason is increasingly used as an indicator of response to nutritional therapy. Reference values for pre-albumin are 16 - 35 mg/dl. A value of dietary intervention is 11 mg/dl because a value below this level means malnutrition. The failure of patients to increase pre-albumin above 11 mg/dl with dietary therapy is an indication that nutritional needs are not met. Concentrations should increase about 1 mg/dl per day or twice a week when the treatment is the appropriate. Non-dietary factors that reduce pre-albumin include stress, inflammation [25-27].

Physical measurements include protein nitrogen balance studies and measurement of creatinine/height index (CHI). Nitrogen balance studies measure the net change in total body protein. An assessment of nitrogen balance can be achieved by measurement of urinary urea (UUN) and compare it with the intake of nitrogen at the same time. The nitrogen balance is calculated as follows: N_2_ = balance intake N_2_ − N_2_ elimination or = [protein (gr)] − (24 hour UUN + 3) [6.25 gr nitrogen]. An "agent" of 3 is added to the equation for nitrogen losses in feces, skin, and the drainage of body fluids. When calculating the nitrogen balance a value of 0 meaning nitrogen balance (healthy adults), nitrogen balance > 0 (protein anabolism exceeds catabolism, usually consistent with pregnancy, growth, and recovery from disease or may indicate nutrient saturation, the goal in nutrition replenishment is a positive nitrogen balance of 4 - 6 grams per day and nitrogen balance < 0 (the protein catabolism exceeds protein anabolism, occurs in situations of famine, increased catabolism due to trauma or surgery, and inadequate nutrition therapy), respectively. CHI measures the 24-hour creatinine excretion in urine and compares with an optimum value based on the ideal weight for height [28].

## Physiopathological Mechanisms of Malnutrition in Acute and Chronic Spinal Cord Injury

### Energy expenditure in spinal cord injury

There is a dramatic increase in energy expenditure, endogenous protein catabolism and nitrogen excretion after injury. Extensive multiple organ trauma, soft tissue injuries and fractures commonly associated with spinal cord injury, may further increase hyper catabolic reactions. Also, after spinal cord injury the body temperature and energy expenditure increases due to pulmonary infections or urinary tract infractions, and pancreatitis. The metabolic rate does not seem to be affected by the small reductions in thyroxin levels in plasma observed after the injury [29-31].

The energy requirements are evaluated in different ways. To determine accurately the early energy expenditure after spinal cord injury, studies compared measurements of real resting energy expenditure (REE) with the Harris-Benedict equation (basic energy expenditure, BEE) [18]. During the first two weeks after the injury, the exact measurements of REE are similar to the estimated calorie needs, when used with BEE stressor/injury factor of 1.6. To avoid overestimation of calorie needs, the deletion of factor activity of 1.2 (rest in bed) is proposed. Kearns et al. reported that in 10 patients, the mean REE after acute injury was only 67% of BEE predicted by Harris-Benedict formula. They hypothesized that non-specific changes in neurogenic stimuli and reduced oxygen consumption by relaxing muscles contributed to their findings. Also, an interesting feature observed is that the REE was raised by 5% with the return of muscle tone [32]. Jeejeboy and Cerra proposed an alternative approach that uses body weight (kg) alone as a determining factor, and omits the variables of age, sex and height as used in HB equation. This type of assessment has proven to be accurate and efficient over time [33, 34]. Ireton-Jones and Owen et al. have developed specific formulas for the obese patient, which is common in SCI subjects. The predefined types may overestimate their needs due to increased fat mass in this population [35, 36].

### Nitrogen balance

Acute post-traumatic nitrogen requirements are much higher than in normal state. The accelerated catabolism of muscle mass results in a supply of amino acids for the acute-phase of protein synthesis, gluconeogenesis, and the healing of wounds. Moreover, administration of glucocorticosteroids after injury may increase the catabolism of protein. The losses of nitrogen in the urine, mainly due to muscle atrophy because of paralysis, are increasing with the severity of the injury. On the other side, Cooper and Hoen stated that the secretion of more than 25 gr/day of nitrogen in the urine during the first two weeks after the injury is insufficient prognostic indicator for functional recovery of paralyzed muscles. The nitrogen losses after an injury are always present and last at least 7 weeks. In cases of acute injury, despite the provision of sufficient quantities of calories and protein usually occurs a negative nitrogen balance (NB), which peaks during the third week after injury. The same phenomenon has been observed in cases of severe poisoning with botulinum toxin (botulism) which resulted in paralysis of muscles. Negative nitrogen balance following injury, has been associated with further findings. During the first weeks after injury, many patients experience a transient positive nitrogen balance, possibly due to initial delays in the loss of nitrogen [37]. Four conscientious objectors were immobilized on pelvic corset and leg casts for 6 to 7 weeks in a metabolic chamber. All 4 subjects showed an increase in nitrogen excretion and negative nitrogen balance. However, it took 4 to 5 days to develop. In conclusion, acute immobilization of paralyzed patients contributes to increased excretion of nitrogen which starts about a week after the injury [38].

### Impaired glucose and lipid metabolism

Glucose and lipid metabolism disrupt in acute post-traumatic phase. Increased hepatic gluconeogenesis and regional response to insulin result in hyperglycemia. The metabolism of glucose in combination with acute nerve injury has been studied extensively, especially as related to ischemia. These studies suggest that hyperglycemia which follows immediately after head injury or spinal cord may worsen the outcome. High serum glucose levels increase the availability of substrate for anaerobic glycolysis, and thus the production of lactic acid, which may have the reverse effect on neurological recovery from injury. The prevention of hyperglycemia, particularly during the first 2 to 8 hours after injury, seems to be very critical for optimal recovery. After 2 to 8 hours after injury, elevated glucose levels may be beneficial, allowing the beginning of intestinal or parenteral feedings in a short time after the injury. It is also likely the serum triglyceride levels to be found elevated due to the accelerated lipogenesis, decreased lipoprotein lipase activity, and impaired clearance of triglycerides [39].

Glucose is the preferred energy molecule for the central nervous system, red blood cells, the cellular tissue, etc. A minimum quantity of 100 - 150 gr glucose per day is required for these functions and prevents the consumption of endogenous protein. The normal rate at which the body metabolizes carbohydrates or glucose is approximately 2 - 4 mg/ Kg/min. In times of severe stress, glucose metabolism may be increased to 3 - 5 mg/Kg/min. In most patients, administration of more than 400 - 500 gr glucose per day, exceeding the body's ability to metabolize and stored as energy. Sources of glucose include not only the liquid diet and peritoneal fluid filtration. Excess glucose is converted into fat (lipogenesis) and leads to an increased ratio of VCO_2_/VO_2_ (or RQ) [40].

The provision of lipids as a source of increased calories can facilitate protein maintenance, reduce the risk of excessive carbohydrates and reduce the total volume of liquid. Lipids are required to account for 30% of total calories supplied. In the acute phase after injury, large amounts of fat, especially as linoleic or omega-6 fatty acids have an immunosuppressive effect by triggering the release of arachidonic acid. This leads to synthesis of prostaglandins and then compresses the delayed hypersensitivity cell-regulated, proliferation of lymphocytes. In the presence of sepsis, high levels of serum triglycerides (250 gr/ml) indicate limited tolerance and decreased need for intravenous fluid delivery. A minimum of 4% of total energy requirements is necessary for the essential fatty acids to avoid deficiencies. Patients with longstanding spinal cord injury are at increased risk for cardiovascular disease and cardiopulmonary disease because of extensive fat intake and limiting activities. The reduced intake of fats and cholesterol is a nutritional counseling intervention in this population to reduce the risks [41].

Deficiencies in zinc and vitamin C have been associated with poor wound healing. The provision of these micronutrients supplementation in patients with SCI with these deficits enhances the healing. Adequate quantities of salts and vitamins are usually provided in a balanced diet. The supplemental micro-nutrient dietary substances are necessary if we suspect shortcomings intake or increased requirements because of circumstances specific diseases. Deficiencies of zinc, vitamin C, vitamin D have been associated with patients with spinal cord injury. Zinc is often prescribed to improve stress ulcers, is known to be involved in structural integrity of collagen. However, zinc levels in serum is similar in patients taking supplements that contain sulfur (220 mg daily) and do not affect the healing process of ulcers sprawling over a period of 2 - 3 months. Opposite physiological effects, such as metabolism of copper, copper deficiency and anemia may be caused by long-term supplementation of large amounts of zinc [42]. The role of vitamin C in collagen synthesis is crucial. Although the supplementation with vitamin C did not accelerate the healing of decubitus ulcers in patients, dietary intake of vitamin C has not been associated with the development of decubitus ulcers. Moreover, given that the subclinical deficiencies are difficult to show up, the minimum recommended dietary intake is proposed to 60mg [43].

Patients with spinal cord injury are prone to developing vitamin D deficiency. Earlier work by Bauman et al suggested that approximately 32% of veterans with spinal cord injury (SCI) were absolutely deficient in vitamin D (25 hydroxyvitamin D [25(OH)D]). Most subjects have a high incidence of vitamin D deficiency as defined by levels of 25(OH)D < 20 ng/mL. The reasons might be due to a combination of low dietary vitamin D intake and avoiding sun exposure because of depression or sensitivity in drugs i.e. dantrolene. In the group of patients with spinal cord injury there was a negative correlation between serum levels of 25-hydroxy-vitamin D and parathyroid hormone. Bone loss increases when there is secondary hyperparathyroidism which complicates the osteoporosis in these patients. For this reason, steps must be taken to correct these disorders [44]. Predisposing factors include also failure to sunlight due to hospitalizations and reduced mobility. There is a direct link between sunlight exposure and vitamin D3 production in the skin, which is biologically inactive but is converted by 25-hydroxylase (25-OHase) enzyme in the liver to 25-OH D, the circulating form of vitamin D. The low intake of vitamin D, which is supplied by food either in vitamin D2 (ergocalciferol, activated ergosterol), found in yeast, or vitamin D3 (cholecalciferol), found in fish, can be bypassed through supplements [45].

## Discussion

### Nutritional deficiency

The average daily dietary needs are modified because of the altered physiology of each body system and psychological integrity of a patient susceptible to an injury, potentially at any age, which cannot exclude the possibility of a pre-existing disease causing nutritional problems [46]. Moreover, the frequent coexistence of spinal cord injury with injuries from other systems, such as brain injury, maxillofacial injuries, fractures, etc., disturbs the normal physiology further. Spinal shock is common after spinal cord injury, can persist for weeks, and is leading to severe hypotension and further complication such as fever, infections, sepsis, and the need for surgery (which may exaggerate the use of body protein for energy-gluconeogenesis) increasing the metabolic rate [47-52]. Studies in malnourished patients stated that malnutrition before a spine stabilization surgery is leading to postoperative complications, hyperthermia, which increases the caloric needs of the patient, and denervation, leading to atrophy and paralysis, which supply amino acids for gluconeogenesis, which, in turn, supplies glucose to meet caloric needs [3]. Metabolic changes are also present with the elevated circulating levels of glucagon, cortisol, catecholamine and cytokines after injury to be primarily responsible for the initial changes in metabolism. Glucose intolerance, which cannot be readily apparent during the acute phase, but may be caused by complications and physiological processes of acute care such as the initial hyper metabolic - catabolic stress response, administration of steroids, the parenteral / enteral nutrition, and atrophy as a consequence of aponeurosis which results in gluconeogenesis [49]. Another serious metabolic issue is negative nitrogen balance, due to excessive secretion of nitrogen because of protein use by the body to meet energy needs in the first week, with a peak at 3 weeks and can last for a period of 7 weeks. This imbalance will respond only slightly increased protein intake and may be non-modifiable as a process during the acute phase. The more severe the injury the greater the amount of nitrogen excreted. Excessive excretion of potassium and abnormal hyponatremia; hypercalcemia, due to immobilization, particularly in young men and hypercalciuria exceed the normal range in 4 weeks, with higher values at 16 weeks, which can persist for a long time. Hypercalcemia occurs with anorexia, abdominal cramps, nausea, vomiting, constipation, polydipsia, polyuria, dehydration and did not respond to diets which restrict the intake of calcium and need to be treated with medication, hydration, and mobilization [49, 53].

### Obesity in spinal cord injury

Obesity in spinal cord injured subjects is mainly central or abdominal obesity leading to metabolic, cardiovascular issues etc. There is conflicting evidence about the contribution of visceral and subcutaneous adipose tissue to different metabolic disorders after SCI. In the past anthropometric measures have been used for the prediction of adipose tissue’s distribution which now days have been replaced by more sophisticated body composition technologies like dual-energy X-ray absorptiometry (DXA), highly precise quantitative techniques such as computerized tomography (CT) and magnetic resonance imaging (MRI) quantifying fat more precise [54, 55]. In a chronic SCI population with paraplegia values of body mass index (BMI, kg/m^2^) were not significant vs. controls, which is a finding in line with the literature [56, 57]. Nevertheless, Gupta et al demonstrated the usefulness of BMI as an indicator of obesity, in body composition in people with spinal cord injury included both tetraplegics and middle-aged people unlike the Greek one which included relatively young individuals [9, 57]. Whether the criteria of BMI may assess obesity in people with spinal cord injury the latest studies show the opposite [58]. In another study 50% of SCI males were overweight by BMI, and more than 10% were classified as obese. Overall, when compared with the general population-observed distribution by BMI, a greater proportion of men with SCI fell into the desirable BMI range and fewer fell into the obese category [59]. These findings show that using the BMI does not contribute substantially in determining the body composition of paraplegics and lowers the percentage of fat in this population, finding that agrees with other studies and shows that the anthropometric measurement with BMI in paraplegics, underestimates fat in body composition when measurements are compared with healthy subjects [60]. Body mass index is a very simple measurement of fat; however it does not distinguish the individual components of weight. The applicability of conventional BMI cut off values is into question [59, 61]. Another critical issue is that the relationship between BMI and disease is typically U- or J-shaped with those in the middle categories of BMI having the lowest risk compared to the lowest extreme and upper levels of BMI. It is under question if the cut-points for underweight, normal, overweight, and obese used in able-bodied populations can be applied to SCI subjects. If we choose percent body fat to measure obesity in SCI it seems that SCI individuals may be at elevated risk; however, more studies are needed to define cut-off points of obesity in SCI subjects and analyzing the impact injury types and duration of injury on the extent of obesity [62, 63].

### Complications of spinal cord injury leading to malnutrition [64]

Serum hemoglobin and hematocrit may reflect a general state of malnutrition. Anemia, defined by low hemoglobin levels (< 14 mg/dl) and hematocrit (< 36%) reduces the oxygen in the blood and impedes the wound healing. Anemia may be due to a preexisting condition or as the result of unbalanced production and distribution of blood cells as a result of reaction to stress, gastrointestinal bleeding or obvious bleeding due to other trauma [65]. Low levels of total serum protein (< 6.4 mg/dl) and protein (< 3.5 mg/dl), accelerate the development of edema, which causes a decrease in skin elasticity and prevent the transfer of oxygen and nutrients from the blood to the skin. Also, the swelling may increase local tissue pressure, causing loss of regional blood flow and tissue damage. The loss of protein and protein secretion in pressure ulcers increases the deficiencies in proteins. Moreover, reduced mobility and immobilization for long period cause pressure ulcers of the skin and the wound but can be prevented by adequate intake of quantity of protein, vitamin E, zinc, and fluids to maintain skin integrity [66].

Pneumonia and paralysis of respiratory muscles through malnutrition may further weaken the respiratory muscles. On the other site excessive feeding may lead to increased oxidation of glucose and production of carbon dioxide to be eliminated and further stress on the respiratory system. The fluid overload or aggressive implementation of parenteral support can lead to pulmonary edema. The reduced hydration can lead to reduced drainage of secretions, atelectasis, and pneumonia. Abdominal distension due to unabsorbed food by mouth or enteral feeding or swallowing air during feeding can lead to compromise the functioning of the diaphragm and predisposes to hypoventilation or aspiration [67]. The paralytic ileus occurs as a result of disturbance of the autonomic and simultaneous or ischemia as a complication of hypokalemia, abdominal trauma or sepsis, generally persists for 72 hours - 1 week and may restrict the movement of the diaphragm [68]. Parenteral nutrition is indicated if persists for more than 3 - 5 days. Ulcers and bleeding, which occur as a result of paralytic vasodilatation with ischemia, steroids, nasogastric tube irritation, and other causes should be treated with oral or enteral feeding as soon as possible but may require parenteral nutrition [69]. Neurogenic bowel requires the right amount of food, fiber and fluids in order to be successful retraining of the bowel, and prevent constipation, diarrhea, incontinence, and autonomic dysreflexia as a result of fecal impaction. Bowel function may be compromised by hyperosmolar feeding through a tube, lactose intolerance or pseudomembranous colitis, prolonged treatment with antibiotics, which can cause diarrhea and require parenteral nutritional support. For neurogenic bladder vitamin C and other supplements are necessary for the acidification of urine and prevention of infection of the urinary tract. The inability of individuals with spinal cord to receive adequate food supply, may be caused by anorexia, early satiety, dysgeusia, smelling problems, immobility, depression, and swallowing disorder i.e. dysphagia or silent aspiration, (caused by a cervical spine stabilization vest like Halo type or other restrictive devices, i.e. a tracheotomy tube, and injuries or nerve palsies and requires parenteral or enteral support, as soon as possible to prevent pressure ulcers), cause anemia and gastrointestinal complications such as stress ulcers, pancreatitis, and reduced mobility. Finally the effect of drugs such as analgesics and barbiturates is crucial.

## Financial

No benefits in any form have been received or will be received from a commercial party related directly or indirectly to the subject of this article.
